# A Novel Combined Model for Predicting Humidity in Sheep Housing Facilities

**DOI:** 10.3390/ani12233300

**Published:** 2022-11-25

**Authors:** Dachun Feng, Bing Zhou, Qianyu Han, Longqin Xu, Jianjun Guo, Liang Cao, Lvhan Zhuang, Shuangyin Liu, Tonglai Liu

**Affiliations:** 1 College of Information Science and Technology, Zhongkai University of Agriculture and Engineering, Guangzhou 510225, China; 2 Smart Agriculture Engineering Technology Research Center of Guangdong Higher Education Institutes, Zhongkai University of Agriculture and Engineering, Guangzhou 510225, China; 3 Guangzhou Key Laboratory of Agricultural Products Quality & Safety Traceability Information Technology, Zhongkai University of Agriculture and Engineering, Guangzhou 510225, China; 4 Academy of Smart Agricultural Engineering Innovations, Zhongkai University of Agriculture and Engineering, Guangzhou 510225, China; 5 Guangdong Province Key Laboratory of Waterfowl Healthy Breeding, Guangzhou 510225, China

**Keywords:** sheep barn, humidity, SVR algorithm, hybrid prediction model

## Abstract

**Simple Summary:**

The hybrid model is proposed to predict humidity in sheep barns, based on a machine learning model combining a light gradient boosting machine with gray wolf optimization and support-vector regression (LightGBM–CGWO–SVR). Influencing factors with a high contribution to humidity were extracted using LightGBM to reduce the complexity of the model, and required hyperparameters in SVR were optimized, adopting the CGWO algorithm to avoid the local extremum problem. The experimental results indicated that the proposed LightGBM–CGWO–SVR model outperformed eight existing models used for comparison on all evaluation metrics; it achieved minimum values of 0.0662, 0.2284, 0.0521, and 0.0083, in terms of MAE, RMSE, MSE, and NRMSE, respectively, and a maximum value of 0.9973, in terms of the R^2^ index.

**Abstract:**

Accurately predicting humidity changes in sheep barns is important to ensure the healthy growth of the animals and to improve the economic returns of sheep farming. In this study, to address the limitations of conventional methods in establishing accurate mathematical models of dynamic changes in humidity in sheep barns, we propose a method to predict humidity in sheep barns based on a machine learning model combining a light gradient boosting machine with gray wolf optimization and support-vector regression (LightGBM–CGWO–SVR). Influencing factors with a high contribution to humidity were extracted using LightGBM to reduce the complexity of the model. To avoid the local extremum problem, the CGWO algorithm was used to optimize the required hyperparameters in SVR and determine the optimal hyperparameter combination. The combined algorithm was applied to predict the humidity of an intensive sheep-breeding facility in Manas, Xinjiang, China, in real time for the next 10 min. The experimental results indicated that the proposed LightGBM–CGWO–SVR model outperformed eight existing models used for comparison on all evaluation metrics. It achieved minimum values of 0.0662, 0.2284, 0.0521, and 0.0083 in terms of mean absolute error, root mean square error, mean squared error, and normalized root mean square error, respectively, and a maximum value of 0.9973 in terms of the R^2^ index.

## 1. Introduction

Sheep farming for meat is a leading industry in the livestock economy of rural inland Northwest China (i.e., Xinjiang), and large-scale intensive farming is the primary mode of production [1]. However, while large-scale farming reduces farming costs, it does involve problems with the physical welfare of the animals, primarily because sheep barns often exhibit high humidity, which tends to breed a great deal of molds and parasites that affect their physiological functions (e.g., reproduction and metabolism) and may lead to various diseases and health problems [2]. Therefore, predicting humidity in sheep barns can provide a theoretical foundation for regulating the barn environment. Commonly used mechanistic prediction models suffer from defects caused by large errors and low accuracy in predicting complex data [3], especially for such humidity data, which typically exhibit complex nonlinear and multi-coupling characteristics, as well as a substantial dynamic time lag [4,5]. Data-driven modeling techniques, based on machine learning and enhanced artificial intelligence, can effectively address these problems, and have been widely discussed in the field of humidity prediction based on time-series data.

As a typical machine learning method, the linear method was discussed earlier in the prediction of pig house environment and humidity. Banhazi et al. [6,7] used a general linear model (GLM) to construct predictive models of environmental factors in pig barns and, subsequently, improved the statistical model using the “leave-one-out” cross-validation technique, which proved helpful for managing pig barn environments. Daskalov [8] used the dynamic discrete autoregressive moving average (ARMA) algorithm to construct an environmental humidity model for pig barns. Even though the linear prediction model was relatively simple, the prediction accuracy of the model was not satisfactory. Other single machine learning methods are often used for univariate rolling prediction of air humidity, for example, Yang et al. [9] developed an air humidity model based on the extended Fourier series model in the least squares method, which overcame the low prediction accuracy of traditional Fourier models. Qadeer et al. [10] proposed the use of a random forest model to predict air humidity, and their prediction results proved that random forest models could accurately predict highly nonlinear data. Although the single variable time series prediction models have high prediction accuracy, the model input parameters do not consider the influence factors of air humidity, which are difficult to apply to the prediction of humidity in livestock houses. In recent years, in addition to traditional machine learning models, the research community has focused on prediction models based on neural networks. Hongkang et al. [11] constructed a dynamic backpropagation neural network (BPNN) model to predict the humidity of a solar greenhouse in northern China, based on recurrent neural networks (RNNs), and took five parameters, such as enumeration and CO_2_ concentration, as input variables. Zou et al. [12] used a convex bidirectional extreme machine-learning-based model to predict the humidity of a solar greenhouse. Wind speed, solar radiation, indoor and outdoor temperature and humidity were used as input variables in the model. Jung et al. [13] used three neural-network-based algorithms, i.e., an artificial neural network (ANN), a nonlinear autoregressive exogenous model (NARX), and a recurrent neural network long short-term memory (RNN-LSTM) to construct greenhouse prediction models for temperature, humidity, and CO_2_; however, the prediction accuracy for humidity was very low for all models. Although these studies on the prediction of greenhouse humidity, based on neural network, provide some convenience for production management, they also have the disadvantage of low prediction accuracy. Besides unsatisfactory prediction accuracy, the above single prediction methods, based on data drive, face other issues, such as poor robustness, the complexity of constructing the models, and difficult selection of neural network parameters in the actual prediction process. In particular, environmental data collected by sensors from large-scale sheep breeding bases are characterized by strong coupling, high dimensionality, nonlinearity, and a large time lag. As a single prediction model considers all the parameters, this approach leads to a complex model structure and poor generalization performance, and, thus, limits the final prediction accuracy. Therefore, the use of a hybrid model to predict such strongly coupled, high-dimensional, and nonlinear parameters has attracted considerable attention and has neem a topic of active research in recent years.

A typical hybrid design helps remove noise, reduce dimensionality, or extract features from high-dimensional input data of models, e.g., wavelet transform and principal component analysis, and, subsequently, uses the obtained data as an input to construct a predictive model using machine learning or deep learning methods. Some hybrid models have been applied to predict humidity and other environmental parameters in livestock houses or greenhouses. For example, Besteiro et al. [14] developed a commercial piglet farm CO_2_ prediction model based on a wavelet neural network designed to consider indoor and outdoor temperatures, indoor humidity, and ventilation rates as input variables. He and Ma [15] extracted four main factors from eight parameters, namely, outside air temperature and humidity, wind speed, and others, based on principal component analysis (PCA) and then constructed a prediction model for indoor greenhouse humidity in north China in winter based on a BPNN model. The test set results showed that the model was not accurate enough. In addition to PCA for feature selection, the light gradient boosting machine (LightGBM) algorithm, developed in recent years [16] as a variant of the improved gradient-boosted decision tree (GDBT), has been widely used as a prediction model in several fields [17,18,19]. Since the heuristic information of the iterative tree can be used as an important measure of features [20], an iterative tree can be constructed using LightGBM to calculate the feature importance measure of a sample to perform feature selection. The built-in exclusive feature bundling algorithm of the LightGBM model compensates for the drawback of PCA that useful information tends to be lost in calculating the dimensionality reduction for less relevant high-dimensional features, and mutually exclusive feature bundling allows for certain cases of non-mutual exclusion among a small number of features, which can better separate redundant features from bundled features to maintaining the accuracy of information filtering. A few works have used LightGBM to reduce the dimensionality of the features of an input model [16,21,22].

In data-driven predictive modeling algorithms, the support-vector regression (SVR) approach rests on a stronger mathematical theoretical foundation than neural-network-based “black box” predictive models [23]. It can effectively solve the problem of constructing high-dimensional data models under limited sample conditions and possesses the advantages of strong generalization ability, convergence to the global optimum, and insensitivity to dimensionality. This method has shown considerable potential to learn nonlinear time-series data, and has been widely used in several fields for prediction, such as late potato blight [24], apricot yields [25], solar radiation [26], and so on.

As the SVR parameter selection is often stochastic and empirical, inappropriate parameter combinations affect the performance and prediction accuracy of such models. Thus, for SVR prediction models, the best parameter combination should be selected to obtain good prediction results. The gray wolf optimization (GWO) algorithm proposed by Mirjalili et al. [27] possesses the advantages of high stability and search capability and has been widely applied to optimize SVR [28,29], BPNN [30] and other models. It has been proven to effectively improve the accuracy and generalization ability of prediction models. As a new metaheuristic optimization technique, GWO possesses stronger global search capability and multimodal function exploration capability than genetic algorithms (GA) and particle swarm optimization (PSO) algorithms [31]. However, it is relatively inefficient and has a tendency to obtain a local optimum [32]. A few researchers have improved the GWO algorithm by introducing new operators, changing control parameters, or updating the GW position. Song et al. [33] proposed a nonlinear model predictive controller, based on the shaded GWO algorithm, to predict the maximum wind energy of wind turbines, and the algorithm was balanced between local and global search by changing the formulae of the gray wolves’ social ranks and position updates to prevent the algorithm from reaching a local optimum. Zhang and Hong [34] improved the search capability and search efficiency of the GWO algorithm by introducing a chaotic tent mapping function to solve the problems of incomplete diversity and premature convergence of wolf packs. Zhao et al. [35] optimized the parameters of a gated recurrent unit (GRU) model using an improved version called IGWO to balance the overfitting and underfitting of the traditional GWO algorithm by improving the objective function. Liu et al. [36] addressed the tendency of the GWO algorithm to fall into local extremes in early stages by introducing a new operator to construct a dynamic variational operator to adjust the difference vector of the wolf pack. Based on these works, and by combining the advantages of other algorithms, in this study, we propose an algorithm called Chaos-GWO (CGWO), based on chaos theory, which is designed to avoid falling into local optima. The proposed approach is further discussed in Section 2.4 and Section 3.3.

Therefore, to improve the lower performance of the existing humidity prediction methods for livestock houses or greenhouses, and overcome the shortcomings of a single SVR model, which may be difficult to adapt to sheep barn humidity prediction, we propose a hybrid prediction model for ambient air humidity in sheep barns based on the proposed combined LightGBM–CGWO–SVR method, to predict the air humidity after 10 min. First, redundant or overlapping information on the environmental features obtained from the sensors is filtered out using the LightGBM model’s built-in dimensionality reduction technique, to avoid excessive computational complexity and overfitting caused by the direct input of multidimensional features. Subsequently, the SVR model hyperparameters are searched and optimized using the GWO algorithm, improved by the chaos operator for better accuracy and reliability.

## 2. Materials and Methods

### 2.1. Data Sources

**Figure 1:**
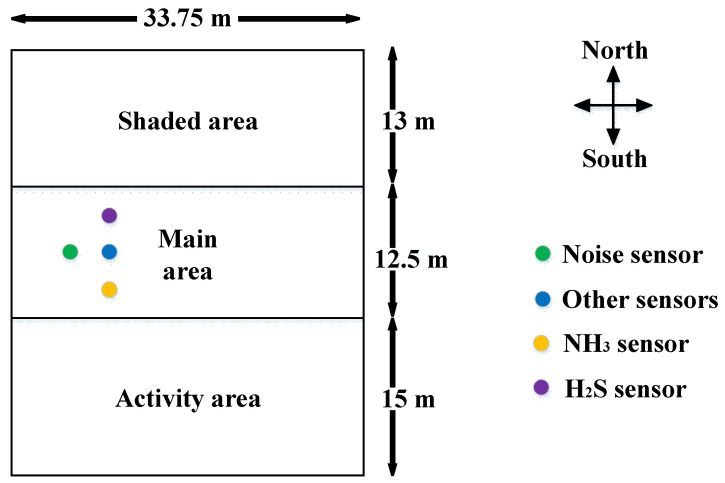
Schematic of the Xinjiang sheep house monitoring.

The source of the data was a sheep breeding facility for meat production located at 44°27′18″ N latitude and 86°10′47″ E longitude in Manas County, Changji Hui Autonomous Prefecture, Xinjiang Uygur Autonomous Region, China. The main space of the facility, with an area of ~422 m^2^, was selected to collect the experimental data. The facility mainly breeds and raises Suffolk sheep, and the sheep barn structure is semi-enclosed and generally divided into three parts, as shown in Figure 1, which included the main area, used as the daily resting area for sheep located in the middle of the barn, a shaded area located in the north, and an eating area located in the south. The main area of the farm was ~33.75 m long and 12.5 m wide, with brick and concrete walls on all sides, windows and doors, a roof made of steel plating, and a floor of compacted soil. In summer, the sheep barn is protected from heat by natural ventilation and a shaded tent on the north side. In winter, the sheep are kept in the main area for warmth and enclosed breeding, and ventilation is provided by exhaust fans. To monitor the environmental parameters of the sheep barn online, temperature, humidity, noise, light, particulate matter 2.5 (PM_2.5_), particulate matter 10 (PM_10_), total suspended particulate (TSP), CO_2_, ammonia, and hydrogen sulfide sensors were deployed in the main area. The ammonia, hydrogen sulfide, noise, and other sensors were installed at 3.2, 3.7, 3.1, and 3.3 m, respectively, from the ground.

### 2.2. Experimental Data Acquisition

**Figure 2:**
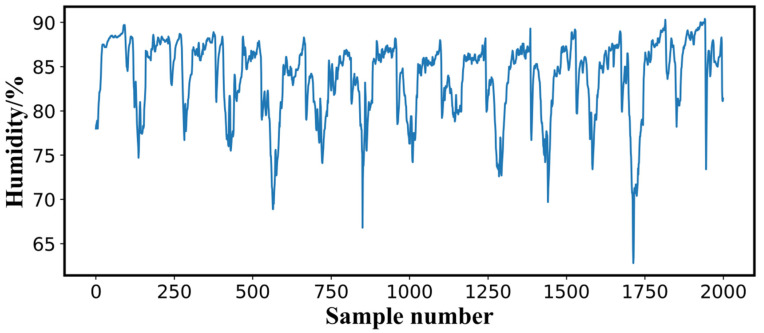
Original nonlinear curve of the values of air humidity.

The sensors installed in the sheep barn performed online data collection at 10-min intervals, and the data were uploaded to a remote IoT cloud service platform through a gateway. The 2000 units of data collected online from 8 to 22 February 2021, were used as the data source for this study; 70% of these data were selected for the training set, and 30% were used for the testing set to train and validate the performance of the proposed humidity prediction model. The raw environmental data are provided in Table 1, and the sequence of the observed air humidity data is illustrated in Figure 2.

### 2.3. Experimental Data Preprocessing

As the parameters in the sheep barn environment had different magnitudes and units, which differed significantly and affected the data analysis and learning results, the influence of magnitudes between parameter sets needed to be eliminated through normalization to enable the algorithms to learn and analyze the hidden structural relations in the data more easily [37]. In addition, standardization was performed to accelerate the training of learning models, and was more stable than an unstandardized training procedure under the same conditions, providing a more uniform fit and better prediction results. The standardization equation is provided as Equation (1):

(1) $${Z_{m}}^{*}=\frac{Z_{m}-\bar{Z_{m}}}{Z_{s}},$$

where $Z_{S}$, $\bar{Z_{m}}$, and ${Z_{m}}^{*}$ denote the standard deviation, mean, and values obtained after normalization, respectively.

### 2.4. Construction of the Combined Forecasting Model for Sheep Barn Humidity

#### 2.4.1. LightGBM for Feature Selection

LightGBM is a lightweight framework implementing a gradient-boosting decision tree, which is an algorithm that converts multiple decision trees into a strong learning machine using weighted linear combinations. It retains samples with large gradients and randomly selects samples with small gradients during training. To counteract the sampling effect in the data distribution, LightGBM introduces a constant multiplier for samples with small gradients in calculating the information gain. For the training set $D:\left\{Y_{1},Y_{2},\cdots ,Y_{n}\right\}$, the negative gradient of the loss function at each iteration corresponds to $\left\{h_{1},h_{2},\cdots ,h_{n}\right\}$, the sampling rate of the large gradient samples is o, and the sampling rate of the small gradient samples is p. The steps used to learn the feature weighting are performed as follows:

Step 1: Sort the sample points in descending order according to the absolute values of their gradients.
Step 2: Select the top o×n samples of the sorted results to generate a subset of the large gradient samples $O_{D}$.
Step 3: Randomly select p×n samples from the remaining samples and generate them as a small gradient sample subset $P_{D}$.
Step 4: Combine the large and small gradient samples ($O_{D}\cup^{\;}P_{D}$) to learn a new decision tree; to calculate the information gain (IG), the small gradient samples are multiplied by a weight factor (1−o)/p, and the information gain of the split feature m for the split point d is calculated as:
  (2) $${\tilde{V}}_{m}\left(d\right)=\frac{1}{n}\left(\frac{{\left(\sum_{Y_{i}\in O_{D},Y_{i,m}\leq d}h_{i}+\frac{1-o}{p}\sum_{Y_{i}\in P_{D},Y_{i,m}\leq d}h_{i}\right)}^{2}}{\sum^{\;}F\left(Y_{i}\right)}+\frac{{\left(\sum_{Y_{i}\in O_{D},Y_{i,m}>d}h_{i}+\frac{1-o}{p}\sum_{Y_{i}\in P_{D},Y_{i,m}>d}h_{i}\right)}^{2}}{\sum^{\;}\left[1-F\left(Y_{i}\right)\right]}\right),$$
  Of which:
  (3) $$F\left(Y_{i}\right)=\{\begin{array}{ll} 1 & Y_{i}\in \left(O_{D}\cup^{\;}P_{D}\right),Y_{i}\leq d\\ 0 & Y_{i}\in \left(O_{D}\cup^{\;}P_{D}\right),Y_{i}>d \end{array}$$
Step 5: Repeat Steps 1–4 for a specified number of iterations or until convergence is reached. After traversing the entire model, the sum of IG of all split nodes for a feature is considered an important weight.

The collected environmental parameters of sheep barns were ranked based on feature importance using LightGBM, and the weights obtained are listed in Table 2. To select strongly correlated features, the key influencing factors of CO_2_ concentration, light intensity, air temperature and PM_2.5_ were screened and applied to the LightGBM model, and the training and testing sets of the Xinjiang sheep barn humidity prediction model were constructed using the screened parameter data.

#### 2.4.2. GWO Algorithm

The GWO algorithm was inspired by the putative hierarchy of Gray Wolves and an associated hunting mechanism. The model is based on the assumption of a strict social hierarchy in a GW pack, which is divided into four levels. The first level is an α wolf, the leader of the pack and responsible for the decision-making of the whole population; the second level is the β wolves, which assist the α wolf in decision-making or other hunting activities; the third level is the δ wolves, which obey the commands of the first two levels and perform tasks, such as scouting and sentry duty; the fourth level comprises ω wolves, which obey the leadership and command of the first three levels. The main components of the GWO algorithm are described below.

(1) Population structure

In the GWO algorithm, each GW represents a potential solution, where the optimal solution is denoted as α, the second best as β, the third best as δ, and ω denotes the remaining candidate solutions and is guided by α, β, and δ in its search.

(2) Surrounding the prey

The GW encircle the prey in the GWO algorithm as described by a mathematical model defined in Equations (4) and (5):

(4) $$D=\left|C\cdot U_{i}\left(n\right)-U\left(n\right)\right|,\;\mathrm{and}$$

(5) $$U\left(n+1\right)=U_{i}\left(n\right)-A\cdot D,$$

where n denotes the current number of iterations, A⋅D denotes the bracketing step, $U_{i}$ denotes the position vector of the prey, and U denotes the current position vector of an individual GW. A and C denote the vectors of random coefficients, which are calculated as given in Equations (6) and (7):

(6) $$A=2a\cdot r_{1}-a,\;\mathrm{and}$$

(7) $$C=2\cdot r_{2},$$

where C varies in the range of (0, 2), which increases the randomness of the distances of other individual GWs from the α, β, and δ wolves, which influences the detection ability of the group and facilitates the GWO algorithm to jump out of local optima. $r_{1}$ and $r_{2}$ denote randomly generated vectors on interval (0, 1), respectively, and a denotes a nonlinear factor that decreases linearly from 2 to 0 with the number of iterations, which is calculated as:

(8) $$a=2-2\left(\frac{n}{n_{max}}\right),$$

where n and $n_{\max}$ denote the current number of iterations and the maximum number of iterations. As the value of a decreases from 2 to 0, A varies in the range of (−a,a). When |A|<1, the GW launches an attack on its prey; that is, an optimal solution is achieved. When |A|>1, the GW has performed a search but abandoned the current prey; thus, this search avoids getting stuck in a local optimal solution and causes the GWO algorithm to continue to search for a solution that is optimal solution.

(3) Hunting process

The α, β, and δ wolves are more aware of the potential location of their prey and occupy an optimal position to attack. Other individual GWs are guided by the α, β, and δ wolves to move toward the prey during the hunting phase, to surround the prey, and to update their positions by moving and repositioning multiple times to eventually reach the prey’s position, respectively. The specific equations for the change in the positions of the GW pack siege are given as:

(9) $$L_{\alpha }=\left|C_{1}\cdot U_{\alpha }\left(n\right)-U\left(n\right)\right|,\;\;L_{\beta }=\left|C_{2}\cdot U_{\beta }\left(n\right)-U\left(n\right)\right|,\;\;L_{\delta }=\left|C_{3}\cdot U_{\delta }\left(n\right)-U\left(n\right)\right|,$$

(10) $$U_{1}=U_{\alpha }-A_{1}\cdot L_{\alpha },\;\;\;\;U_{2}=U_{\beta }-A_{2}\cdot L_{\beta },\;\;\;\;U_{3}=U_{\delta }-A_{3}\cdot L_{\delta },\;\mathrm{and}$$

(11) $$U_{i}\left(n+1\right)=\frac{U_{1}+U_{2}+U_{3}}{3},$$

where $L_{\alpha },\;L_{\beta },\;\mathrm{and}\;L_{\delta }$ denote the distances of the individual GWs from the α, β, and δ wolves, respectively; $U_{\alpha },\;U_{\beta },\;\mathrm{and}\;U_{\delta }$ denote the current positions of α, β, and δ wolves, respectively; $U_{1},\;U_{2},\;\mathrm{and}\;U_{3}$ denote the updated positions of the individual GWs toward α, β and δ wolves, respectively; and $U_{i}\left(n+1\right)$ denotes the mean of the updated positions of the individual GWs along the directions of α, β, and δ wolves.

#### 2.4.3. Improvement of GWO Based on Chaotic Operators

(1) Improvements of update mechanism based on random coefficient vectors C

For GWO, exploration and exploitation capabilities are crucial to optimal performance and mainly depend on the value of the stochastic exploration coefficient C. Thus, choosing a suitable value of C to coordinate the exploration and exploitation capabilities of GWO is critical.

Inspired by chaos theory, we introduced time-varying acceleration constants in $C_{1}$, differential mean perturbed time-varying parameters in $C_{2}$, and sigmoid-based acceleration coefficients in $C_{3}$ to enhance the randomness of C. Compared with random search, a chaotic algorithm could help in a thorough search with a higher probability and speed. It overcomes the tendency of the GWO algorithm to fall into a local optimum, owing to existing performance, and distributes the search more uniformly to obtain better global convergence performance and faster convergence. The improved equations, $C_{1}$, $C_{2}$, and $C_{3}$, are defined as:

(12) $$C_{1}=0.5+0.5\cdot \exp\left(-\frac{n}{500}\right)+1.4\cdot \frac{sin\left(n\right)}{30},$$

(13) $$C_{2}=1+1.4\cdot \left(1-\exp\left(-\frac{n}{500}\right)\right)+1.4\cdot \frac{sin\left(n\right)}{30},\;\mathrm{and}$$

(14) $$C_{3}=\frac{1}{\left(1+\exp\left(-\frac{0.0001\cdot n}{n_{max}}\right)\right)\left(0.5-2.5\right){\left(\frac{n}{n_{max}}\right)}^{2}},$$

where n and $n_{\max}$ denote the current iteration number and maximum iteration number, respectively. Equations (12)–(14) are used instead of Equation (7).

(2) Algorithm implementation of CGWO

In summary, the implementation process of the proposed CGWO algorithm is given as follows:

Step 1: Initialize the algorithm parameters of the GW population size M, with a maximum number of iterations $n_{max}$.
Step 2: Population initialization. Randomly generate M individual GWs $\left\{\;U_{i},i=1,2,\cdots ,M\right\}$, iterate through all the individual wolves, calculate the fitness values of these M individual GWs $\left\{\;f\left(U_{i}\right),i=1,2,\cdots ,M\right\}$, and identify α, β, and δ wolves: $U_{\alpha },\;U_{\beta },\;\mathrm{and}\;U_{\delta }$. Initialize the nonlinear decreasing factor a and random exploration vectors A and C.
Step 3: Set n=1 (current number of iterations).
Step 4: If $n<n_{max}$ (or when the iteration condition is satisfied).
Step 5: Update the distances and directions of the movement of individual GWs in the current pack from the α, β, and δ wolves using Equations (9)–(11), respectively, and update the location of each individual GW using Equations (4) and (5).
Step 6: Update the random exploration vectors A, C, and the convergence factor a using Equations (6)–(8).
Step 7: Calculate the fitness of each individual GW for the current iteration $\left\{\;f\left(U_{i}\left(n\right)\right),i=1,2,\cdots ,M\right\}$, and compare it with the fitness of the last iteration. If the new fitness is higher than the current fitness, the new fitness replaces the current fitness as the global optimal fitness and updates the wolf’s position. If the new fitness value is not higher than the current fitness, it remains unchanged.
Step 8: Update α, β, and δ wolves: $U_{\alpha },\;U_{\beta },\;\mathrm{and}\;U_{\delta }$.
Step 9: n=n+1, return to Step 4 when the iteration condition is satisfied, exit the loop when the maximum number of iterations ($n_{max}$) is reached, obtain the position of the α wolf, which is the best value for the SVR parameters C, g, and ε, and the algorithm ends.

#### 2.4.4. SVR

For a given sample training set $\left(m_{i},n_{i}\right),i=1,2,\cdots ,N$, m denotes the independent variable in the sample training set, n is the dependent variable in the sample training set and N is the number of samples. The SVR is linearly fitted by the nonlinear feature mapping function ϕ(m), which maps the data m onto a high-dimensional linear space. The linear function of SVR in the high-dimensional feature space can be expressed as:

(15) f(m)=μϕ(m)+b,

where μ denotes the normal vector and b denotes the bias term.

To control the accuracy of the fit, an insensitive loss function ε is introduced. If the difference between the predicted value f(m) of m and the actual value n is less than ε, the predicted value does not suffer any loss. Otherwise, the predicted value is considered to have suffered a loss, and the optimization model is expressed in Equation (16):

(16) $$F_{\varepsilon }\left(f\left(m\right),n\right)=\{\begin{array}{ll} 0 & \left|f\left(m\right),n\right|<\varepsilon \\ \left|f\left(m\right)-n\right|-\varepsilon & \left|f\left(m\right),n\right|\geq \varepsilon \end{array}.$$

Penalty coefficients C were introduced for error-free fitting at positive and negative relaxation levels, i.e., $\tau_{i}$ and $\tau_{i}{}^{*}$, respectively, with the minimum of the sum of the complexity of the regression function and the fitting error as the final optimization objective, as expressed in Equation (17):

(17) $$min\frac{1}{2}{||\mu ||}^{2}+C\sum_{i=1}^{N}(\tau_{i}+\tau_{i}{}^{*}),$$

(18) $$s.t.\{\begin{array}{c} n_{i}-\mu \phi \left(m_{i}\right)-b\leq \varepsilon +\tau_{i}{}^{*}\\ \mu \phi \left(m_{i}\right)+b-n_{i}\leq \varepsilon +\tau_{i} \end{array}\;\;\;\;\tau_{i},\tau_{i}{}^{*}\geq 0\left(i=1,2,\cdots ,N\right).$$

Using the Lagrange multipliers $\lambda_{i}$ and $\lambda_{i}{}^{*}$ such that the partial derivatives of the variables μ, b, $\tau_{i}$, and $\tau_{i}{}^{*}$ are zero, the abovementioned functions were transformed into the pairwise problem of SVR, as expressed in Equation (19):

(19) $$\{\begin{array}{c} max\frac{1}{2}\overset{N}{\underset{i=1}{\sum }}\overset{N}{\underset{j=1}{\sum }}\left(\lambda_{i}-\lambda_{i}{}^{*}\right)\left(\lambda_{j}-\lambda_{j}{}^{*}\right)\cdot \kappa \left(m_{i},m_{j}\right)-\varepsilon \overset{N}{\underset{i=1}{\sum }}\left(\lambda_{i}+\lambda_{i}{}^{*}\right)+\overset{N}{\underset{i=1}{\sum }}m_{i}\left(\lambda_{i}-\lambda_{i}{}^{*}\right)\\ s.t.\{\begin{array}{l} \overset{N}{\underset{i=1}{\sum }}\left(\lambda_{i}-\lambda_{i}{}^{*}\right)=0\\ \lambda_{i},\lambda_{i}{}^{*}\in \left[0,C\right] \end{array} \end{array},$$

where $\kappa \left(m_{i},m_{j}\right)$ denotes the kernel function, which implies that the inner product is calculated after mapping $m_{i}$ and $m_{j}$ onto a high-dimensional feature space.

By solving the pairwise problem, the SVR regression function could be expressed as:

(20) $$f\left(m\right)=\sum_{i=1}^{N}\left(\lambda_{i}-\lambda_{i}{}^{*}\right)\kappa \left(m_{i},m_{j}\right)+b$$

In this experiment, the radial basis kernel function RBF, which is currently widely used, was considered, and expressed as:

(21) $$\kappa \left(m_{i},m_{j}\right)=\exp\left(-\frac{||m_{i}-m_{j}||{}^{2}}{g^{2}}\right),$$

where g denotes the width parameter of the radial basis kernel function.

#### 2.4.5. Humidity Prediction Model of Sheep Barns Based on LightGBM-CGWO-SVR

**Figure 3:**
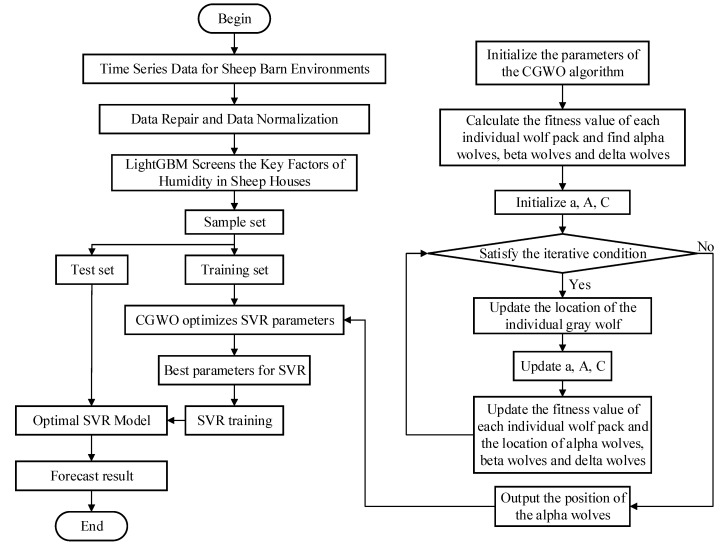
Flowchart of the LightGBM–CGWO–SVR algorithm.

A hybrid prediction model. based on the proposed LightGBM-CGWO-SVR method, was implemented for sheep barn humidity (Figure 3). The specific algorithmic steps are as follows:

Step 1: Perform data repair on the collected data.
Step 2: Use LightGBM to calculate the IG for the sheep barn environmental data after the data have been restored, and each parameter factor has been ranked in terms of feature importance, selecting strongly correlated features.
Step 3: Normalize the data after dimensionality reduction, based on LightGBM, and divide the training and test sets in a ratio of 7:3.
Step 4: Based on the training set, optimize C, g, and ε for the SVR model using the CGWO algorithm described in Section 2.4.3.
Step 5: Apply the best hyperparameters obtained using CGWO, that is, C, g, and ε, to the SVR model to obtain the combined LightGBM–CGWO–SVR prediction model.

## 3. Results and Discussion

### 3.1. Experimental Setup and Parameter Determination

The experimental calculations were performed using a computer with an 11th Gen Intel(R) Core (TM) i7-1165G7 processor at 2.80 GHz and 16 GB of memory, running the Windows 10 64-bit operating system with the Anaconda3 platform and the Python 3.6 programming langue (64-bit). The simulation environment was completed by installing the NumPy, Sklearn, and LightGBM packages.

The CGWO parameters were set as follows. The size of the wolf pack was set to 20, the random exploration vectors A and C were set to 20, and the SVR parameters were searched in the range of [0.01, 10]. SVR uses RBF, and the optimal combination of hyperparameters was obtained by searching the SVR model globally using the CGWO algorithm. The maximum number of iterations was 20, although the fourth iteration started to converge in the experiment.

### 3.2. Performance Criteria

In this study, the coefficient of determination (R^2^), mean absolute error (MAE), root mean square error (RMSE), normalized root mean square error (NRMSE), and mean squared error (MSE) were used as evaluation metrics to computationally assess the prediction results.

(1) R^2^ shown in Equation (1) was used to assess the fit between the predicted and actual humidity, and considered a value in the range of (0, 1), a value closer to 1 represented a better fit between the predicted and actual humidity values.
  (22) $$R^{2}=\frac{\sum_{k=1}^{r}{\left(f_{k}^{\prime }-\bar{f}\right)}^{2}}{\sum_{k=1}^{r}{\left(f_{k}-\bar{f}\right)}^{2}}.$$

(2) MAE was the average distance between the individual and predicted humidity data; a smaller MAE implied that the predicted value was closer to the actual humidity data (Equation (23)).
  (23) $$MAE=\frac{1}{r}\sum_{k=1}^{r}\left|f_{k}-f_{k}^{\prime }\right|$$

(3) MSE represented the mean of the square of the difference between individual humidity data and the predicted humidity data. RMSE was the root mean square result of the MSE, which could be intuitive, in terms of the order of magnitude performance; normalized RMSE converted the RMSE value within (0, 1). The smaller the MSE, RMSE, and NRMSE, the smaller the error between the predicted and true values, and the more accurate the model. They were expressed as:
  (24) $$RMSE=\sqrt{\frac{1}{r}\sum_{k=1}^{r}{\left(f_{k}-f_{k}^{\prime }\right)}^{2}},$$
  (25) $$NRMSE=\frac{\sqrt{\frac{1}{r}\sum_{k=1}^{r}{\left(f_{k}-f_{k}^{\prime }\right)}^{2}}}{\bar{f}},\;\mathrm{and}$$
  (26) $$MSE=\frac{1}{r}\sum_{k=1}^{r}{\left(f_{k}-f_{k}^{\prime }\right)}^{2}$$

In the equations above, $f_{k}$, $f_{k}^{\prime }$, r, and $\bar{f}$ denoted the original data value, predicted value of $f_{k}$, amount of data, and average value of $f_{k}$, respectively.

### 3.3. Results and Analysis

**Figure 4:**
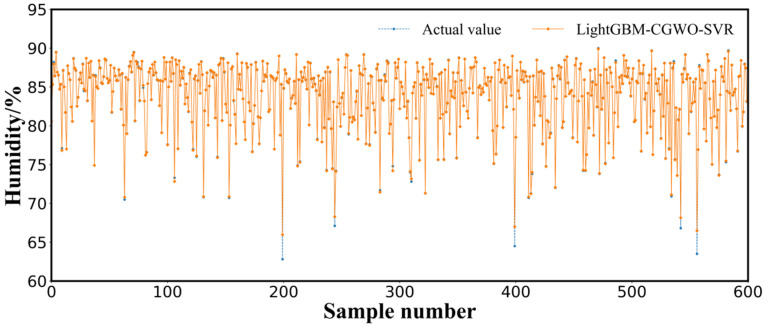

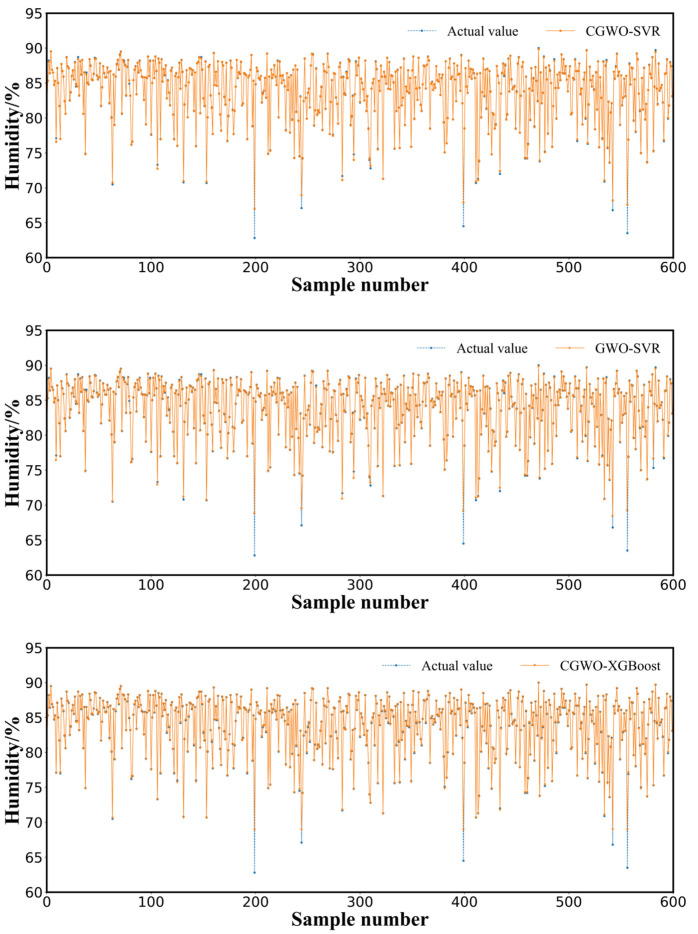

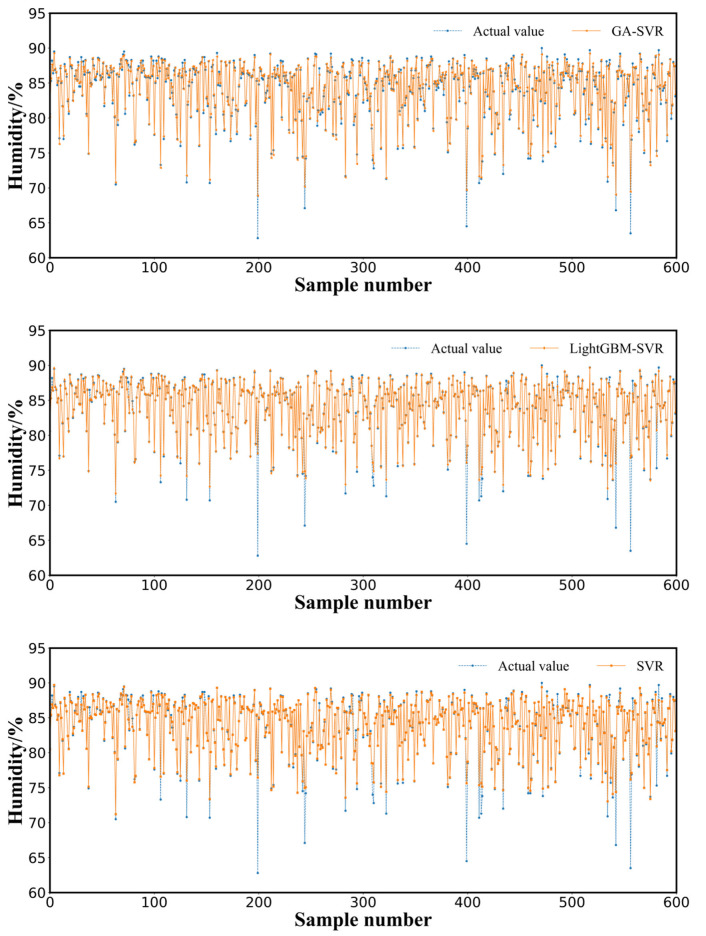

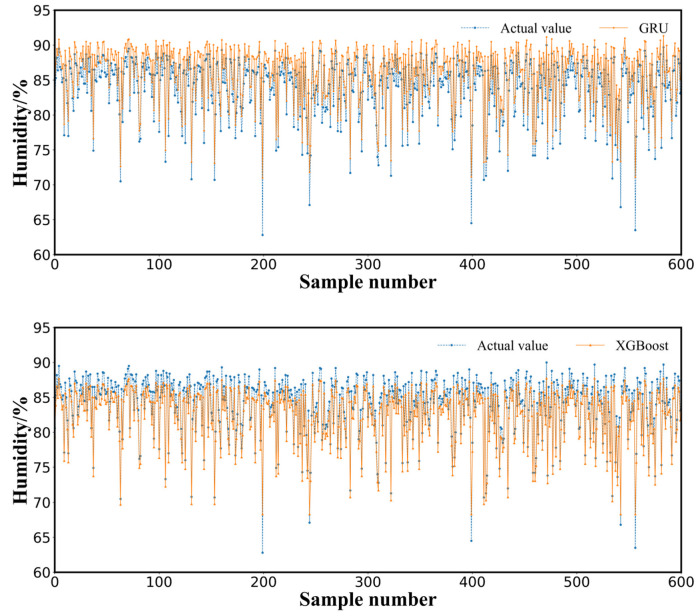
Measured versus computed humidity (validation level).

To demonstrate the superior performance and effectiveness of the proposed LightGBM–CGWO–SVR model in predicting the humidity of sheep barn facilities, our approach was compared with the following models: GRU, SVR, extreme gradient boosting (XGBoost), GA-SVR, GWO-SVR, CGWO-XGBoost, CGWO-SVR, and LightGBM-SVR. These were computed using the same training and validation datasets, and parameters of different SVR models are shown in Table 3. The prediction performance for 10 min details of the different models on the final validation dataset are listed in Table 4. The real humidity measurements on the validation set with the predicted values of each model are shown in Figure 4. According to the results of the comparison presented in Table 4, the evaluation indices of the proposed LightGBM-CGWO-SVR-based sheep barn humidity prediction model were better than those of the other prediction models. The minimum values of MAE, RMSE, MSE, and NRMSE of the model were 0.0662, 0.2284, 0.0521, and 0.0083, respectively, among all the models, and R^2^ achieved the highest value of 0.9973. As shown in Figure 4, the prediction curve of the LightGBM–CGWO–SVR model was able to significantly fit the true value curve, indicating that the LightGBM–CGWO–SVR-based sheep barn humidity prediction model exhibited high accuracy and fitting performance.

**Figure 5:**
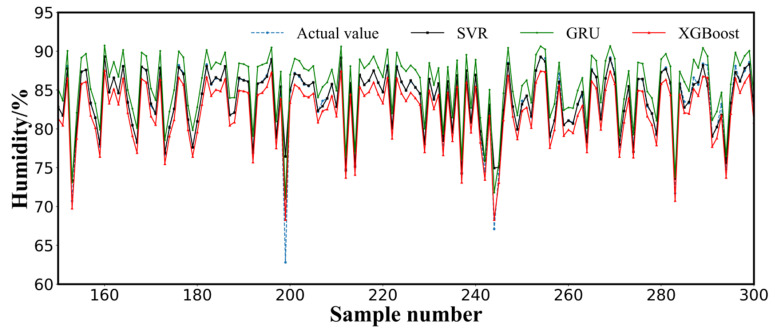
Measured versus computed humidity of GRU, XGBoost, and SVR (part of validation data).

For a single model, the prediction accuracy of the SVR model was higher than that of the GRU and XGBoost models. The MAE, RMSE, MSE, and NRMSE values of the SVR model were reduced by 76%, 30%, 51%, and 30%, respectively, compared with those of GRU, and 76%, 19%, 34%, and 19%, respectively, compared with those of XGBoost. Compared with GRU and XGBoost, the R^2^ of SVR improved by 9% and 4%, respectively. This indicated that the SVR model exhibited good adaptability to sheep barn humidity data. The real observed values of the local humidity and the predicted values of the GRU, XGBoost, and SVR models are shown in Figure 5. The prediction curves of the SVR model were closer to the real values than those of the GRU and XGBoost models, particularly for the prediction curves of the GRU model, which fluctuated more than those of the XGBoost and SVR models. The values of the GRU and XGBoost models deviated more from the predicted value when the humidity value was approximately 85%, whereas SVR achieved better coverage. This might have occurred because GRU models exhibit issues of gradient disappearance or explosion for increasing amounts of data and model volume, and XGBoost suffers from the difficulty of tuning parameters to cope with high-dimensional nonlinear features. In contrast, SVR models, the computational complexity of which is not highly correlated with the dimensionality of the input parameters, demonstrate better generalization ability and prediction accuracy when dealing with high-dimensional data.

**Figure 6:**
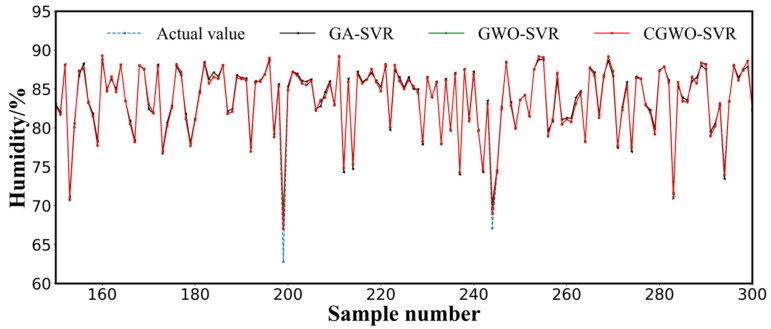
Measured versus computed humidity of GA–SVR, GWO–SVR, and CGW–SVR (part of validation data).

When the SVR algorithm hyperparameters were globally searched and optimized using three different search algorithms (i.e., GA, GWO, and CGWO), the SVR algorithm optimized using CGWO exhibited lower MAE, RMSE, MSE, and NRMSE values of 80%, 43%, 73%, and 48%, respectively, compared to the SVR algorithm optimized using GA, whereas R^2^ was improved by 1%. Compared with the GWO-optimized SVR algorithm, the MAE, RMSE, MSE, and NRMSE values were reduced by 4%, 30%, 48%, and 28%, respectively, and R^2^ was improved by 0.4%. Thus, CGWO outperformed GA and GWO in terms of optimization and could maintain a good balance between local and global searches. Moreover, the real measurement values of humidity localized in the validation set were compared with the predicted values obtained from the GA–SVR, GWO–SVR, and CGWO–SVR models (Figure 6). Using the optimization algorithm to perform a global search of the SVR hyperparameters significantly improved the prediction performance of the SVR model, and the CGWO-optimized SVR model was able to fit the original curve more ideally. The result was closer to the true values in the prediction of peaks than the models optimized by GA and GWO. Compared with GA, the GWO algorithm introduces nonlinear decreasing factors and random coefficient vectors to increase the randomness of the distance between other GW individuals and the head, which improved the detection ability of the group. The results obtained in this study, by introducing chaos operators into the GWO optimization algorithm to form the CGWO optimization algorithm, further validated that chaos operators can overcome the shortcomings of the GWO algorithm’s tendency to fall into local optima and its low efficiency in path planning.

**Figure 7:**
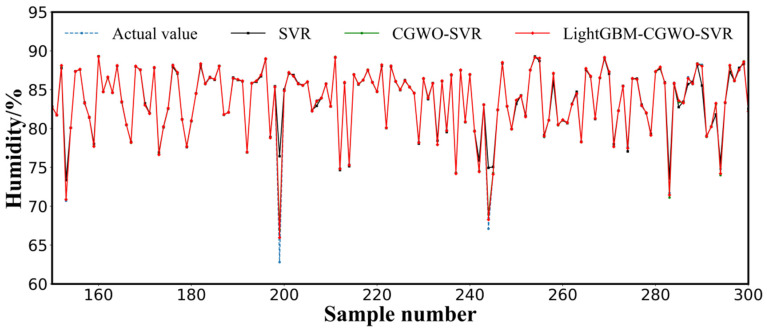
Actual versus computed humidity of SVR, CGW–SVR, and LightGBM–CGWO–SVR (part of validation data).

The data in Table 4 indicate that the MAE, RMSE, MSE, and NRMSE of the SVR model optimized by CGWO were reduced by 5%, 24%, 43%, and 24%, respectively, compared with those of the model optimized by CGWO with XGBoost, which further demonstrated the superior fitting ability of SVR for nonlinear problems. The MAE, RMSE, MSE, and NRMSE of the LightGBM–CGWO–SVR model obtained by further reducing the dimensionality of the data using LightGBM on top of the CGWO–SVR model were reduced by 9%, 25%, 44%, and 26%, respectively, compared with those of CGWO–SVR. This demonstrated that feature extraction of sheep barn data using LightGBM could reduce the input dimensionality of the model, filter redundant information and irrelevant features, reduce the size of the algorithm, and enhance the generalization performance of the model. In addition, the MAE, RMSE, MSE, and NRMSE of the LightGBM–SVR model obtained using LightGBM to reduce the dimensionality were 25%, 3%, 6%, and 3%, respectively, lower than those of the single SVR model, which implied that using LightGBM to reduce the dimensionality of the data could improve the accuracy of the model. This was supported by the comparison of the local datasets predicted by the single SVR, CGWO–SVR, and LightGBM–CGWO–SVR models with respect to humidity (Figure 7).

**Figure 8:**
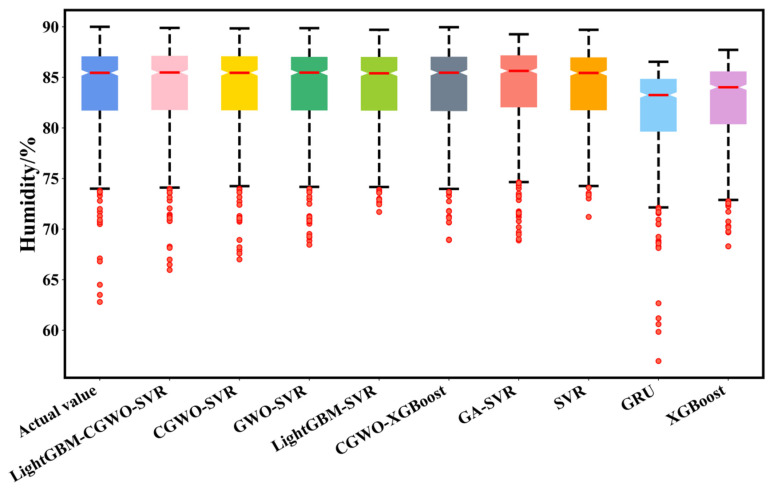
Box schemes of actual versus computed humidity (validation level).

**Figure 9:**
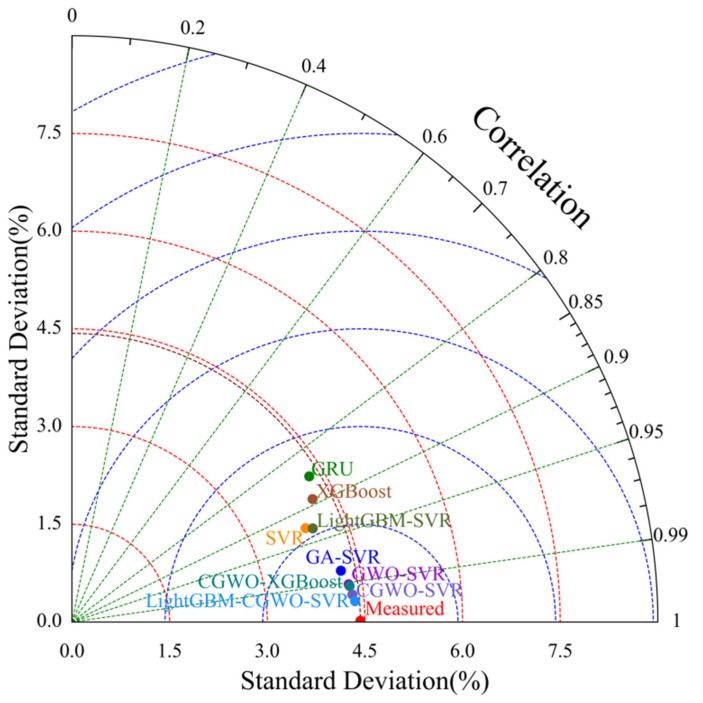
Taylor schemes of actual versus computed humidity (validation level).

Figure 8 shows the box plot scheme of the predicted moisture value spatial diffusion at the validation level, based on different models (i.e., GRU, SVR, XGBoost, GA–SVR, GWO–SVR, CGWO–XGBoost, CGWO–SVR, and LightGBM–SVR), where GRU and XGBoost predictions on the validation set faced difficulties in matching the actual true observations. The predictions of the LightGBM–CGWO–SVR model were more stable, with the upper quartile, lower quartile, and median close to the actual values and agreeing with the actual observations over the entire range (e.g., maximum and minimum). The predictions of the LightGBM–CGWO–SVR model were more accurate than those of the other models for the offset values that appeared in the original environmental data. The Taylor plots of the accuracy of the predicted humidity values of each model on the validation set compared with the actual values are shown in Figure 9, where each point represents the error statistic of the predicted humidity value of a model compared with the actual value. Among them, the LightGBM–CGWO–SVR model was closest to the arc where the actual values were located, and had the shortest distance from the actual values, indicating that the model possessed the smallest root mean square error between the predicted and actual values. Moreover, the Taylor plot shows that the proposed model exhibited the highest correlation coefficient compared to the other models.

In addition, autocorrelations of the observed time series data are often an important factor affecting prediction performance. Table 5 shows the accuracy results of the model predicted by the proposed LightGBM–CGWO–SVR, which used 10-, 30-, 60-, and 90-min lag time datasets, respectively. The results showed the accuracy of the model developed slowly decreased over time. Despite all this, the observed humidity data with a lag of less than 100 min were sound information, and the prediction accuracy of the dataset with a lag of 90 min also showed good performance. Its R^2^ reached 0.9940, which was only 0.39% less than that of the dataset with a lag of 10 min.

### 3.4. Discussion

In a previous related study, He and Ma [15] obtained good performance on the training set using the BPNN and PCA combination forecasting model, with R^2^ of 09768 and RMSE of 1.6745. However, the test set results with R^2^ of 0.8842 showed that the model generalization ability was still insufficient. The RNN–LSTM prediction model developed by Hongkang et al. [11] achieved good performance to temperature in the greenhouse, but it was not satisfactory to humidity prediction, and the R^2^ was only 0.82 for the dataset with a 10 min lag.

The proposed LightGBM–CGWO–SVR prediction model exhibited high prediction accuracy for the collected time-series data. It could help accurately predict changes in humidity pattern in sheep barn facilities at intervals of 10 min or even 90 min. Furthermore, it could effectively adapt to nonlinear and high-dimensional data on humidity in sheep barns and exhibited superior generalization ability and prediction accuracy, compared to advanced algorithms, such as GRU and XGBoost models. It is possible to apply constructed combination models to predict and regulate air humidity in intensive sheep farming barns.

It should be noted that the data was acquired in February, when Xinjiang is in winter and very cold. For this reason, the sheep house was not always strongly ventilated, but ventilated intermittently and regularly. That is to say, a large part of the humidity data in this data set was not significantly affected by ventilation. Of course, the ventilation factor should not be ignored in model construction for the warm season, when the sheep house would be frequently ventilated.

## 4. Conclusions

The final simulation results of the proposed LightGBM–CGWO–SVR model and a comparison with other advanced models implied the following points:

(1) The characteristics of the environmental factors of the sheep barn were screened using LightGBM without considering environmental variables with small effects on humidity. The key influencing factors of CO_2_ concentration, light intensity, air temperature, and PM_2.5_ were finally screened from the nine variables, including air humidity, CO_2_ concentration, PM_2.5_, PM_10_, light intensity, noise, TSP, NH_3_ concentration, and H_2_S concentration, and used for modeling, which effectively improved the computational efficiency of the model and its accuracy.
(2) The CGWO algorithm obtained by introducing chaotic operators in this study retained the advantages of a simple structure, few control parameters, and straightforward implementation of the GWO algorithm, while improving the global search capability of the traditional GWO algorithm and solving the problem of randomness and empiricality in the selection of SVR parameters. The optimized SVR algorithm exhibited higher prediction accuracy and better generalization ability than an SVR algorithm optimized using GA and GWO.
(3) The performance of the proposed LightGBM–CGWO–SVR model in predicting the humidity for 10 min in a sheep barn facility exceeded that of the GRU, SVR, XGBoost, GA–SVR, GWO–SVR, CGWO–XGBoost, CGWO-SVR, and LightGBM–SVR models. The minimum values of the MAE, RMSE, MSE, and NRMSE indices were 0.0662, 0.2284, 0.0521, and 0.0083, respectively, and R^2^ achieved the highest value of 0.9973. The results indicated that the proposed model can provide an effective guide for the accurate prediction of humidity in sheep barns in for Suffolk sheep, in Xinjiang, and could provide a reference for future research on the prediction of other environmental factors, such as NH_3_ and H_2_S concentration.

## Figures and Tables

**Table 1:** Experimental original data collected from 8 to 22 February 2021.

Time	Air Temperature /°C	Air Humidity /%	CO_2_ /(mL/m^3^)	PM_2.5_ /(μg/m^3^)	PM_10_ /(μg/m^3^)	Light Intensity /lx	Noise /dB	TSP /(μg/m^3^)	NH_3_ /(mL/m^3^)	H_2_S /(mL/m^3^)
2021/2/8 17:11:56	4.2	78	1355	16.3	81.3	146	31.1	120.7	0	5.2
2021/2/8 17:21:35	3.9	78.4	1330	26.5	107.1	97	72.7	165.3	0	5.2
......	......	......	......	......	......	......	......	......	......	......
2021/2/22 17:12:15	6.6	81.2	855	12.5	24	97	40.2	45.3	0	5.2
2021/2/22 17:22:15	6.4	81.4	780	9.3	24.6	183	34.1	42	0	5
2021/2/22 17:32:16	6.3	81.4	770	12.2	34.8	159	31.8	58.2	0	1.4

**Table 2:** Parameter feature importance ranking based on the LightGBM model.

Parameter	Feature Weights
CO_2_	29.741152
Light intensity	13.195553
Air temperature	13.092886
PM_2.5_	6.540839
H_2_S	5.084741
PM_10_	3.862321
Noise	3.290505
TSP	1.477563
NH_3_	0.000000

**Table 3:** Parameters of different SVR models in experiment.

Prediction Model	C	g	ε
SVR	1.000	0.100	0.100
GA–SVR	6.564	0.831	0.011
GWO–SVR	8.958	0.023	0.016
CGWO–SVR	9.346	0.011	0.040
LightGBM–SVR	1.000	0.100	0.100
LightGBM–CGWO–SVR	9.849	0.010	0.059

**Table 4:** Experimental results of different prediction models.

Prediction Model	Error Type				
	MAE	RMSE	MSE	NRMSE	$R^{2}$
GRU ^1^	1.4307	1.7019	2.8964	0.0625	0.8522
SVR	0.3451	1.1878	1.4110	0.0436	0.9280
XGBoost ^2^	1.4372	1.4645	2.1450	0.0538	0.8906
GA–SVR	0.3732	0.5897	0.3477	0.0216	0.9822
GWO–SVR	0.0763	0.4230	0.1789	0.0155	0.9908
CGWO–XGBoost ^3^	0.0772	0.4025	0.1620	0.0147	0.9917
CGWO–SVR	0.0731	0.3048	0.0929	0.0112	0.9952
LightGBM–SVR	0.2596	1.1545	1.3328	0.0424	0.9320
LightGBM–CGWO–SVR	0.0662	0.2284	0.0521	0.0083	0.9973

^1^ learning_rate = 0.079, subsample = 0.5, n_estimators = 50. ^2^ learning_rate = 0.659, subsample = 0.5, n_estimators = 50. ^3^ learning_rate = 0.08, batch_size = 256, nb_epoch = 500.

**Table 5:** Comparison of LightGBM–CGWO–SVR prediction accuracy at time steps of 10, 30, 60, and 90 min for humidity.

	MAE	RMSE	MSE	NRMSE	R^2^
10 min	0.0597	0.1950	0.0380	0.0073	0.9979
30 min	0.0674	0.2494	0.0622	0.0092	0.9972
60 min	0.2181	0.2786	0.0776	0.0149	0.9956
90 min	0.2254	0.3298	0.1087	0.0179	0.9940

## Data Availability

Not applicable.
